# Alteration of 11**β**-Hydroxysteroid Dehydrogenase Type 1 and Glucocorticoid Receptor by Ethanol in Rat Liver and Mouse Hepatoma Cells

**DOI:** 10.1155/2013/218102

**Published:** 2013-06-01

**Authors:** Zhaojie Meng, Xueying Bao, Ming Zhang, Shengnan Wei, Wenguang Chang, Jing Li, Li Chen, B. L. Grégoire Nyomba

**Affiliations:** ^1^Department of Pharmacology, School of Norman Bethune Medical Sciences, Jilin University, Changchun, Jilin 130021, China; ^2^The 208th Hospital of the Chinese People's Liberation Amry, Changchun, Jilin 130062, China; ^3^Department of Internal Medicine, University of Manitoba, Winnipeg, MB, Canada R3E3P4

## Abstract

Alcohol is a potential risk factor of type 2 diabetes, but its underlying mechanism is unclear. To explore this issue, Wistar rats and mouse hepatoma cells (Hepa 1–6) were exposed to ethanol, 8 g*·*kg^−1^ *·*d^−1^ for 3 months and 100 mM for 48 h, respectively. Glucose and insulin tolerance tests *in vivo* were performed, and protein levels of 11*β*-hydroxysteroid dehydrogenase type 1 (11*β*-HSD1) and glucocorticoid receptor (GR) in liver and Hepa 1–6 cells were measured. Alterations of key enzymes of gluconeogenesis phosphoenolpyruvate carboxykinase (PEPCK) and glucose 6 phosphatase (G6Pase), as well as glycogen synthase kinase 3a (GSK3**α**), were also examined. The results revealed that glucose levels were increased, and insulin sensitivity was impaired accompanied with liver injury in rats exposed to ethanol compared with controls. The 11*β*-HSD1, GR, PEPCK, G6Pase, and GSK3**α** proteins were increased in the liver of rats treated with ethanol compared with controls. Ethanol-exposed Hepa 1–6 cells also showed higher expression of 11*β*-HSD1, GR, PEPCK, G6Pase, and GSK3**α** proteins than control cells. After treatment of Hepa 1–6 cells exposed to ethanol with the GR inhibitor RU486, the expression of 11*β*-HSD1 and GR was significantly decreased. At the same time the increases in PEPCK, G6Pase, and GSK3**α** levels induced by ethanol in Hepa 1–6 cells were also attenuated by RU486. The results indicate that ethanol causes glucose intolerance by increasing hepatic expression of 11*β*-HSD1 and GR, which leads to increased expression of gluconeogenic and glycogenolytic enzymes.

## 1. Introduction

During several decades, many cohort studies from the medical epidemiology literature have observed a close association between ethanol consumption and type 2 diabetes [1, 2]. Some studies have suggested that heavy drinking induces the development of type 2 diabetes and is a potential risk factor for diabetes; however, consuming moderate amounts of alcohol has been reported to reduce the incidence of diabetes [3]. So far, the relationship between alcohol and diabetes has not been well characterized. Notably, the mechanism of alcohol-induced diabetes remains uncertain.

Type 2 diabetes is a metabolic syndrome characterized by insulin resistance and decreased in insulin secretion [4, 5]. Full-blown type 2 diabetes is preceded by impaired glucose tolerance (IGT) and impaired fasting glucose (IFG) globally termed prediabetes, which is associated with an increased risk for the development of type 2 diabetes [6, 7]. Subjects with IFG have increased hepatic glucose output and early dysfunction of insulin secretion, while subjects with IGT have moderate-to-severe insulin resistance in the muscle [8, 9]. It is well known that phosphoenolpyruvate carboxykinase (PEPCK) and glucose 6 phosphatase (G6Pase) are the rate-limiting enzymes in hepatic gluconeogenesis, whereas glycogen synthase kinase 3 (GSK3) plays an important role in glucose production and storage [10–13].

As antagonists of insulin action, glucocorticoids are major sources of increased glucose production in type 2 diabetes though upregulation of key enzymes in gluconeogenesis. Excess tissue glucocorticoid action may contribute to the hyperglycemia and insulin resistance associated with type 2 diabetes. Inactive glucocorticoids (cortisone, 11-dehydrocorticosterone) are converted into active forms (cortisol, corticosterone) by 11**β**-hydroxysteroid dehydrogenase type 1 (11**β**-HSD1) [14]. Active glucocorticoids bind to glucocorticoid receptor (GR), stimulate the expression of PEPCK and G6Pase, and enhance glucose production from both gluconeogenesis and glycogen degradation in liver [15].

It is well established that long-term excessive ethanol consumption impairs glucose tolerance, induces insulin resistance, and leads to the development of type 2 diabetes. Ethanol causes oxidative and endoplasmic reticulum stress in pancreatic **β** cells [16, 17], and this can result in impairment of insulin secretion [18]. In the present study, we investigated whether glucose tolerance is altered in association with 11**β**-HSD1 and GR in rats chronically treated with high amounts of ethanol corresponding to human chronic alcoholism.

## 2. Materials and Methods

### 2.1. Materials

Dulbecco's Modified Eagle's Medium (DMEM), fetal bovine serum (FBS), penicillin/streptomycin, and 0.25% trypsin EDTA solution were purchased from Gibco BRL (Grand Island, NY, USA). RU486 was purchased from Sigma (St. Louis, MO, USA). Insulin was purchased from Eli Lilly, Changchun, China. Glucose oxidase kit was obtained from Beijing BHKT Clinical Reagent Co., Beijing, China. [^125^I]Insulin radioimmunoassay kit was purchased from Tianjin Nine Tripods Medical & Bioengineering Co., Tianjing, China. Polyclonal antibodies to 11**β**-HSD1, GR, PEPCK, G6Pase, GSK3*α* and actin, and goat anti-rabbit IgG horseradish peroxidase conjugate were all purchased from Santa Cruz Biotechnology (Santa Cruz, CA, USA). GAPDH was purchased from Epitomics (Burlingame, CA, USA). ECL Western Blotting Substrate was purchased from Pierce (Thermo Fisher Scientific, Rockford, USA). Chemical reagents for western blot were obtained from Sigma and polyvinylidene difluoride membranes were from Bio-Rad (Hercules, USA).

### 2.2. Animal Experiments

Male Wistar rats (200–220 g) obtained from the Experimental Animal Holding Facility of Jilin University were randomly divided into two groups: normal control group and ethanol-treated group. After one week of acclimatization, the ethanol group was given 36% ethanol (8 g*·*kg^−1^ *·*d^−1^) via an intragastric tube, and the control group was given an equal volume of water. This administration was carried out twice daily at 9 AM and 4 PM for three months. Both groups of rats were given free access to a normal chow and water. Body weight and food intake were recorded weekly. The protocols for animal care and handling were approved by the Animal Care and Use Committee of Jilin University.

### 2.3. Cell Culture

Mouse hepatoma (Hepa 1–6) cells obtained from ATCC were grown in DMEM supplemented with 10% fetal bovine serum, 1% L-glutamine (200 mM), penicillin (40 units*·*mL^−1^), and streptomycin (40 *μ*g*·*mL^−1^). Hepa 1–6 cells were passaged using a 0.25% trypsin-EDTA solution, seeded at 1 × 10^6^ cells*·*dish^−1^ in 3 mL DMEM with 10% FBS, and incubated at 37°C for 48 h. Dishes of cells were then randomly divided into 4 groups: (1) control, (2) control + RU486, (3) ethanol, and (4) ethanol + RU486. The cells in ethanol and ethanol + RU486 groups were treated with 100 mM ethanol refreshed every 12 h for 48 h. 10 *µ*M of RU486, an inhibitor of GR, was added to the cells at the 24th h of ethanol incubation in control + RU486 and ethanol + RU486 groups for 24 h. RU486 was dissolved in ethanol to a stock concentration of 10 mM, which was diluted 1000 times with the culture media, and the same concentration of the solvent was used for control and ethanol groups.

### 2.4. Intraperitoneal Glucose Tolerance Test (IPGTT)

IPGTT was conducted at 3 months of age after a 16 h fast using an i.p. glucose injection (2 g*·*kg^−1^). Blood was taken by tail snip at 0, 30, 60, and 120 min after the glucose injection. Glucose concentration in serum samples was determined using a glucose oxidase kit.

### 2.5. Insulin Tolerance Test (ITT)

ITT was performed at 3 months of age after a 12 h fast using an i.p. insulin injection (0.75 U*·*kg^−1^). Blood was obtained by the same method as for IPGTT to measure glucose concentration.

### 2.6. Plasma Insulin

The rats were fasted for 16 h and blood was taken from the abdominal aorta under anesthesia with i.p. injection of urethane (1 g/kg body weight). Insulin concentration in each sample was measured by RIA, and plasma glucose concentration was determined using a glucose oxidase kit. 

### 2.7. Western Blotting

Liver tissue and Hepa 1–6 cells were homogenized at 4°C in 1 mL or 500 *μ*L of ice cold TES buffer (20 mM Tris-HCl, pH 7.4, containing 250 mM sucrose, 1 mM EDTA, 1 mM phenylmethylsulfonyl fluoride, 0.01 mM leupeptin, and 5 *μ*g*·*mL aprotinin) for 60 min, and the lysate was centrifuged at 10,000 rpm for 5 min at 4°C. Aliquots of the supernatant were removed for protein analysis by the Bradford method (Bio-Rad). The samples (160 *μ*g proteins) were denatured by boiling for 5 min and separated by 10% SDS polyacrylamide gel electrophoresis and then electroblotted onto a polyvinylidene difluoride membranes (Bio-Rad) at 4°C. After blocking in 5% (w/v) nonfat milk for 2 h at room temperature, the membranes were incubated with respective rabbit polyclonal specific primary antibodies with gentle agitation overnight at 4°C. The membranes were washed 3 times for 10 min each with 15 mL of TBST (10 mM Tris-HCl, 150 mM NaCl, and 0.1% (v/v) Tween-20) and then incubated with a secondary antibody (1 : 2000 goat anti-rabbit IgG horseradish peroxidase conjugate) at room temperature for 2 h. The bands of proteins were visualized with ECL on a X-ray film. The protein bands were scanned and quantified using the Quantity One image analysis software (Bio-Rad).

### 2.8. Statistical Analysis

All data were expressed as the mean ± SEM. Statistical analyses were performed using *t*-test for significance using SPSS software (version 13.0 for Windows). *P* < 0.05 was considered to be significant.

## 3. Results

### 3.1. Effects of Ethanol on Body Weight and Metabolic Parameters in Rats

Mean body weight and food intake during the study period are summarized in Table 1. Body weight was not significantly different between control and ethanol groups. Average food intake of the 12 weeks in ethanol group (67.5 ± 0.53 g*·*kg^−1^ *·*d^−1^ [403.3 ± 3.17 cal*·*kg^−1^ *·*d^−1^]) was slightly lower compared with controls (74.3 ± 1.03 g*·*kg^−1^ *·*d^−1^ [443.9 ± 6.15 cal*·*kg^−1^ *·*d^−1^]). However, the amount of ethanol ingested (8 g*·*kg^−1^ *·*d^−1^) provided 47.8 cal*·*kg^−1^ *·*d^−1^, increasing the total caloric intake in Ethanol group to 451.1 cal*·*kg^−1^ *·*d^−1^, which is similar to that of control group. Metabolic changes induced by ethanol are presented in Table 2. Ethanol-treated group had higher fasting blood glucose, total cholesterol, triglyceride, alanine aminotransferase, and aspartate aminotransferase levels compared with the control group (*P* < 0.05–0.01). Plasma insulin levels were reduced in ethanol-treated group (*P* < 0.05).

**Figure 1:**
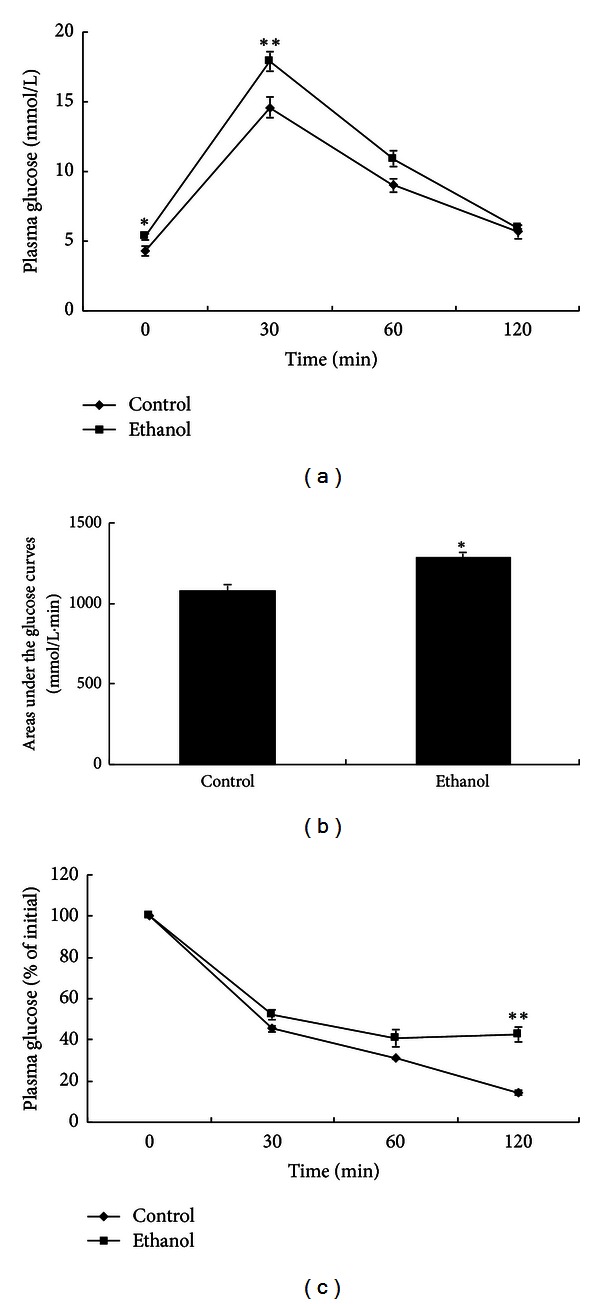
Glucose and insulin tolerance after 3 months of ethanol intake (8 g*·*kg^−1^ *·*d^−1^). Plasma glucose concentrations during intraperitoneal glucose tolerance test (IPGTT), the area under the IPGTT glucose curve, and the glucose concentrations during insulin tolerance test (ITT) are shown as the mean ± S.E.M (*n* = 20). *P < 0.05, **P < 0.01 ethanol versus control.

IPGTT and ITT were carried out in ethanol and control groups to more accurately determine glucose tolerance and insulin sensitivity (Figure 1). As shown by the IPGTT glucose curve, rats of the ethanol group had higher blood glucose compared with the control group, and the areas under the glucose curves (mmol*·*L^−1^ *·*min) were significantly greater in the ethanol-treated group compared with controls (*P* < 0.05). During insulin tolerance test (ITT), the glucose concentration declined slowly in ethanol-treated group, and at 120 min the glucose level (percentage of initial) was clearly higher in the ethanol group than in the control group (*P* < 0.01). This result demonstrated that 3 months of ethanol intake (8 g*·*kg^−1^ *·*d^−1^) caused insulin resistance. Overall these data indicate that long-term ethanol intake can result in insulin resistance, glucose intolerance, and alteration of lipid metabolism.

### 3.2. Effects of Ethanol on 11**β**-HSD1 and GR Proteins in the Rat Liver

**Figure 2:**
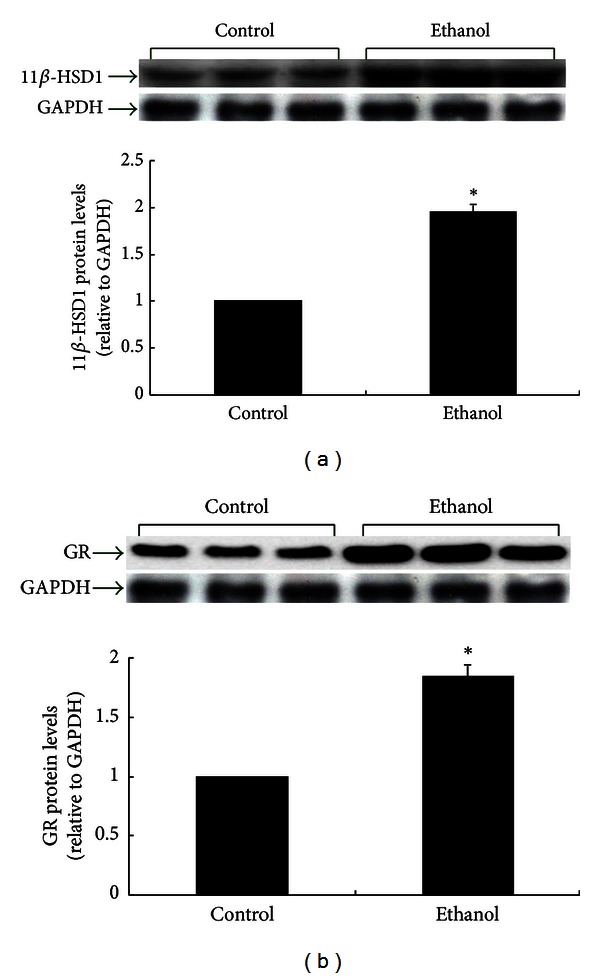
11**β**-HSD1 and GR proteins in the liver of control and ethanol rats after 3 months of ethanol intake (8 g*·*kg^−1^ *·*d^−1^). Protein levels are expressed relative to the control and shown as the mean ± SEM (*n* = 6). *P < 0.05 ethanol versus control.

To investigate the alterations of 11**β**-HSD1 and GR in liver of rats after ethanol exposure, their protein levels were determined using western immunoblot (Figure 2). The protein level of 11**β**-HSD1 was significantly elevated in the liver of ethanol-treated rats compared with controls (Figure 2, *P* < 0.05). At the same time, the protein expression of GR was higher in the ethanol than in the control group (Figure 2, *P* < 0.05).

### 3.3. Effects of Ethanol on Major Gluconeogenic Enzymes and Glycogen Synthase Kinase 3 in Rat Liver

**Figure 3:**
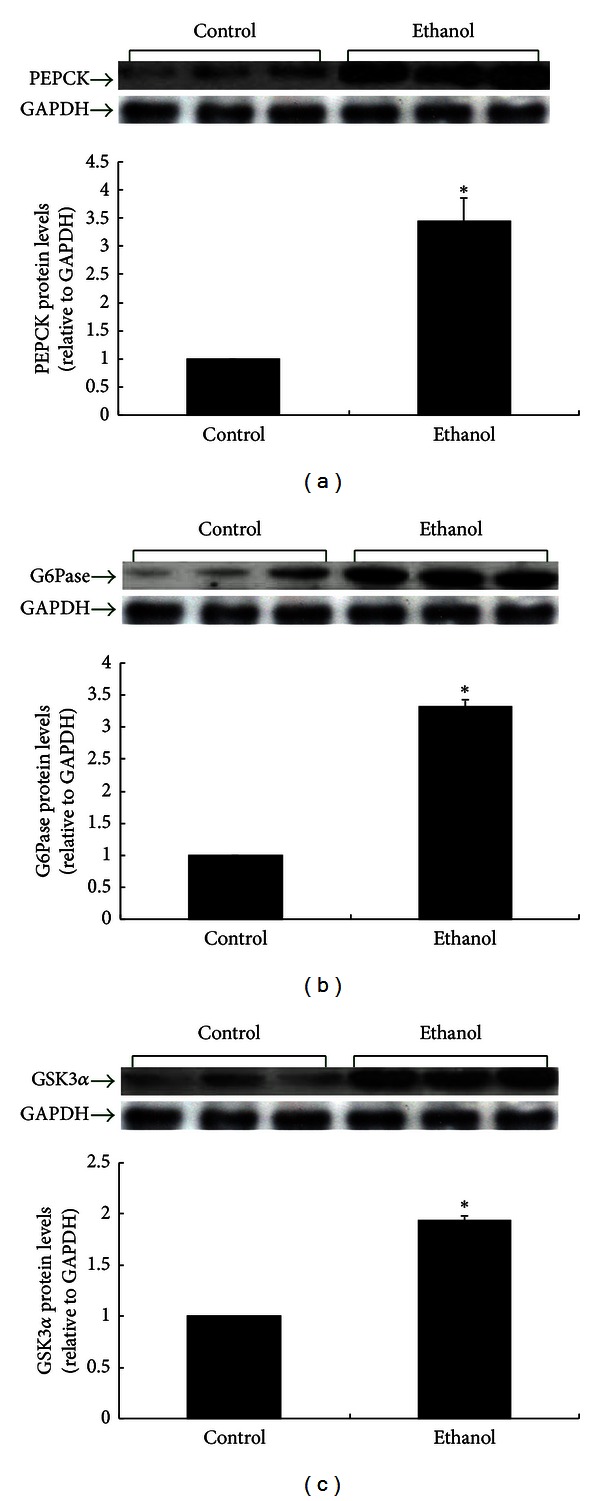
PEPCK, G6Pase, and GSK3*α* proteins in the liver of control and ethanol rats after 3 months of ethanol intake (8 g*·*kg^−1^ *·*d^−1^). Protein levels are expressed relative to the control and shown as the mean ± SEM (*n* = 6). *P < 0.05 ethanol versus control.

The expression of PEPCK and G6Pase, two rate-limiting enzymes in gluconeogenesis, was significantly increased in ethanol-treated rats compared with controls (Figure 3, *P* < 0.05), explaining at least in part the hyperglycemia of ethanol-treated rats. As well, the level of GSK3*α* was higher in ethanol-treated rats than in controls (Figure 3, *P* < 0.05). GSK3 inactivates glycogen synthase, which is the rate-limiting enzyme in glycogen synthesis, and overexpression of GSK3 decreases glycogen synthesis in liver and impairs glucose utilization.

### 3.4. Effects of Ethanol on 11**β**-HSD1 and GR in Hepa 1–6 Cells

**Figure 4:**
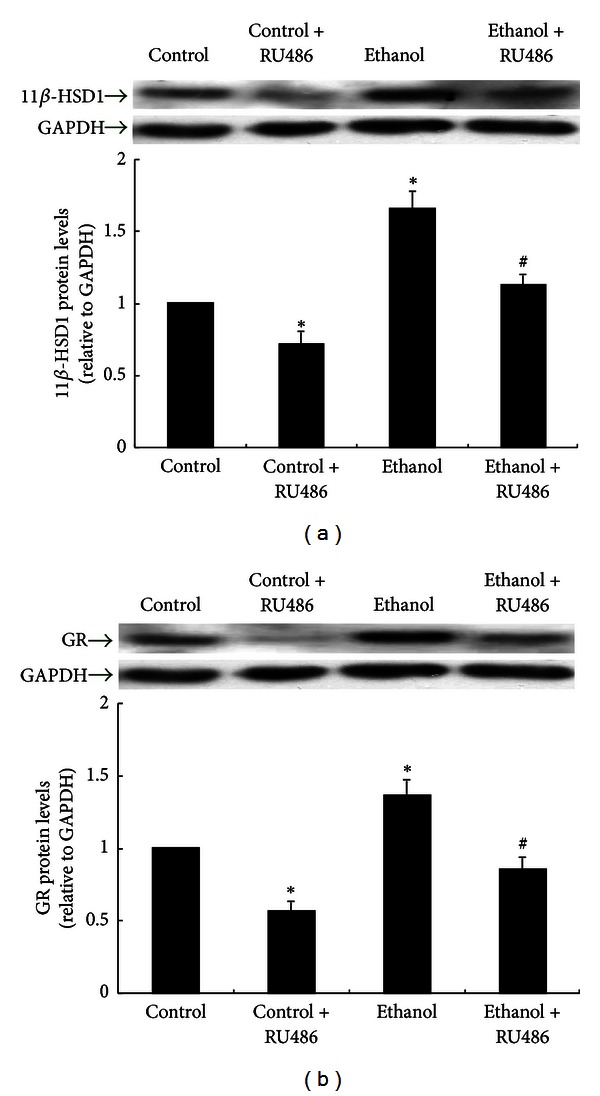
11**β**-HSD1 and GR proteins in Hepa 1–6 cells. Groups of cells were treated with 100 mM ethanol and/or 10 *µ*M RU486, as described in Section 2. Protein levels are expressed relative to control and shown as the mean ± SEM. (*n* = 6). *P < 0.05 versus control; ^#^ *P* < 0.05 versus ethanol.

Liver is one of the major organs responsible for glucose metabolism; therefore, we further examine if the observations in adult rats occur also in the hepatic cells (Hepa 1–6). Hepa 1–6 cells were treated with 100 mM ethanol refreshed every 12 h for a total of 48 h. After 24 h of ethanol treatment, 10 *µ*M RU486 was added. Preliminary results using MTT assay demonstrated that Hepa 1–6 cell viability was not altered by this concentration of RU486 (data not shown). The protein level of 11**β**-HSD1 was significantly elevated in Hepa 1–6 cells treated with ethanol compared with control cells (Figure 4). The GR inhibitor RU486 remarkably reduced the protein expression of 11**β**-HSD1 in both control and ethanol-treated cells. The GR protein levels showed similar alterations (Figure 4).

### 3.5. Effects of Ethanol on Gluconeogenic Enzymes in Hepa 1–6 Cells

**Figure 5:**
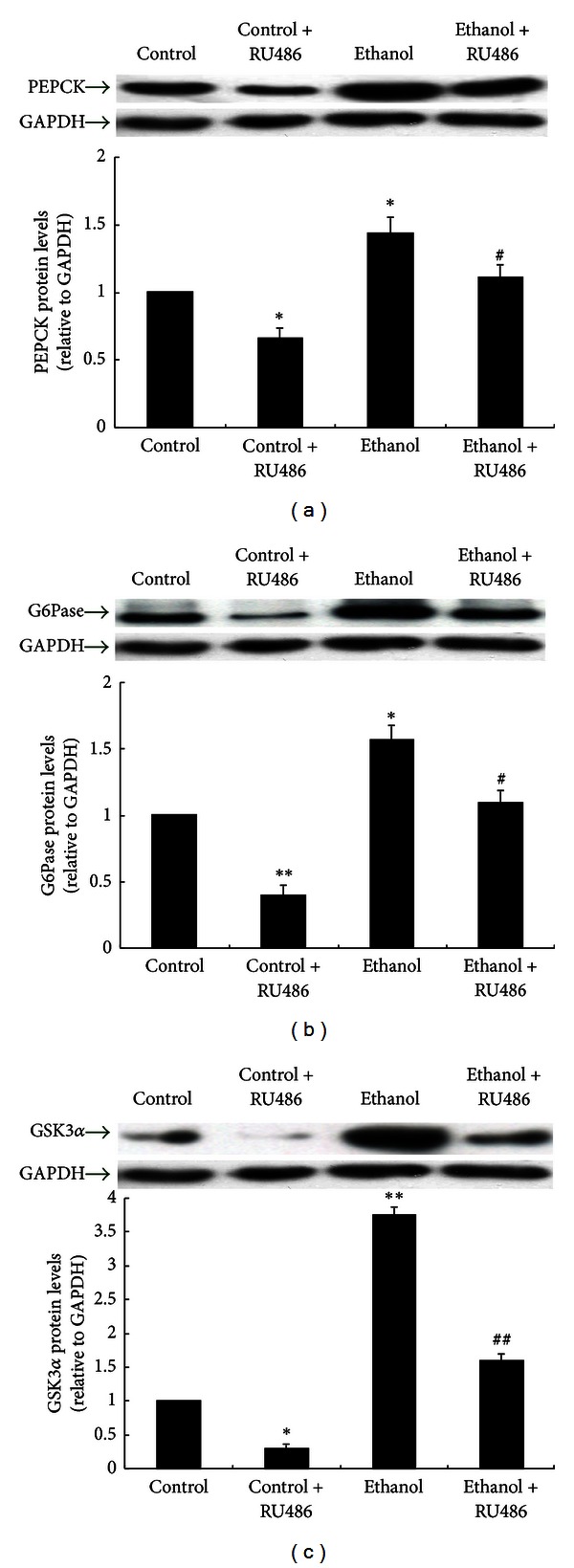
PEPCK, G6Pase, and GSK3*α* proteins in Hepa 1–6 cells. Groups of cells were treated with 100 mM ethanol and/or 10 *µ*M RU486. Protein levels are expressed relative to the control and shown as the mean ± SEM. (*n* = 6). *P < 0.05, **P < 0.01 versus control; ^#^ *P* < 0.05 versus ethanol.

As observed in rat liver *in vivo*, the PEPCK protein level was significantly higher in ethanol-treated than control Hepa 1–6 cells (*P* < 0.05). In these cells, RU486 decreased the PEPCK protein expression (Figure 5). The protein expression of G6Pase presented similar changes (Figure 5).

### 3.6. Effect of Ethanol on Glycogen Synthase Kinase in Hepa 1–6 Cells

As shown in Figure 5, GSK3*α* protein level was markedly increased in Hepa 1–6 cells treated with ethanol, and RU486 reduced the GSK3*α* protein expression in Hepa 1–6 cells with or without prior ethanol treatment. 

## 4. Discussions

Heavy ethanol consumption is a potential risk factor for type 2 diabetes. Human drinking alcohol at doses of 50–60 g*·*kg^−1^ twice per day develops type 2 diabetes [19, 20]. In the present study, rats given ethanol at 8 g*·*kg^−1^ *·*d^−1^ for 3 months had glucose intolerance and reduced insulin sensitivity in association with altered lipid regulation. The rats also had reduced fasting insulin levels, consistent with the suggestion that excessive ethanol causes pancreatic **β** cell dysfunction and apoptosis through oxidative and endoplasmic reticulum stress [16, 17]. The associations of elevated fasting glucose and insulin resistance suggest that ethanol causes alterations of glucose regulation leading to both IFG and IGT. Given these characteristics, the focus of the present research was on the effect of ethanol on enzymes regulating hepatic glucose metabolism, as these could both explain insulin resistance and elevated fasting glucose. First, the rate-limiting enzymes in hepatic gluconeogenesis and glycogen synthesis involved in the development of type 2 diabetes were determined in rats exposed to ethanol. The results showed that alcohol consumption increased expression of PEPCK and G6Pase, which are key enzymes of gluconeogenesis. In addition, ethanol enhanced the protein expressions of hepatic GSK3*α*, one isoform of glycogen synthase kinase 3 (GSK3). GSK3 is a constitutively active kinase in resting cells that becomes rapidly inactivated by phosphorylation at Ser 21 (GSK3*α*) and Ser 9 (GSK3b) in response to insulin [21]. Both GSK3 expression and activity are elevated in muscle and liver tissues of diabetic humans and rodents [22, 23]. Moreover, GSK3 inhibitors improve insulin sensitivity in rodent models of diabetes, alleviating hyperglycemia by decreasing hepatic gluconeogenesis and stimulating glycogen synthesis [24, 25]. Therefore, the present study indicates that elevated expression of PEPCK, G6Pase, and GSK3*α* may be implicated in etiology of glucose intolerance and type 2 diabetes induced by long-term heavy alcohol consumption. 

Accumulating evidence suggests that PEPCK and G6Pase are regulated by 11**β**-HSD1 and GR via amplification of glucocorticoid action within the tissue [15]. 11**β**-HSD1, as NADPH-dependent reductase, converts inactive cortisone (11-dehydrocorticosterone in rats) into active cortisol (corticosterone). Enhanced 11**β**-HSD1 activity results in the production of excess tissue glucocorticoids, which bind and induce local GR activation which is associated with visceral obesity and type 2 diabetes [14, 26]. It has been shown that pharmacological blockade of 11**β**-HSD1 expression prevents the generation of active glucocorticoids and reduces hepatic GR expression, which in turn results in the suppression of both PEPCK and G6Pase mRNA expression and improvement of insulin resistance in diabetic *db/db *mice and obese Zucker rats [27]. In addition, GR blockade with RU486 attenuated the phenotype of type 2 diabetes through the inhibition of the expression of GR and 11**β**-HSD1 in the liver [28]. Corticosterone-induced expressions of GR, 11**β**-HSD1, and PEPCK were also abolished by RU486 [29]. These published data indicate the existence of a positive relationship between GR and 11**β**-HSD1 in regulation of hepatic gluconeogenic enzymes, implicating GR or 11**β**-HSD1 as a potential target for the treatment of type 2 diabetes and obesity. The present study showed that 11**β**-HSD1 and GR protein levels were significantly increased in rats and Hepa 1–6 cells exposed to ethanol, whereas the 11**β**-HSD1 and GR protein levels were depressed in Hepa 1–6 cells after RU486 treatment. RU486 also reduced the protein expression of PEPCK, G6Pase, and GSK3*α*, which are regulated by 11**β**-HSD1 and GR. Therefore, the data suggest that elevated 11**β**-HSD1 and GR may contribute to the increased expression of PEPCK, G6Pase, and GSK3*α* in the liver of ethanol-treated rats. 

In summary, ethanol-exposed rats have impaired glucose tolerance. The protein expression of enzymes involved in liver gluconeogenesis (PEPCK, G6Pase) and glycogen synthesis (GSK3*α*) was increased in rats exposed to alcohol in association with an upregulation of 11**β**-HSD1 and GR. GR blockade with RU486 reversed all these anomalies. The results indicate that elevated 11**β**-HSD1 and GR, which increase gluconeogenesis and reduce glycogen synthesis, may contribute to the development of glucose intolerance in rats chronically consuming high amounts of alcohol.

## Figures and Tables

**Table 1:** Body weight and food intake of ethanol and control rats (mean ± SEM, *n* = 20).

Week	Body weight (g)		Food intake (g·kg^−1^·d^−1^)	
	Control	Ethanol	Control	Ethanol
1	213 ± 5.4	214 ± 5.9	73.2 ± 2.34	66.4 ± 2.80
2	242 ± 5.8	239 ± 6.2	71.0 ± 2.89	68.2 ± 1.26
3	269 ± 7.1	254 ± 7.7	69.5 ± 1.85	68.5 ± 2.36
4	288 ± 4.6	277 ± 7.0	71.2 ± 2.43	69.7 ± 1.08
5	305 ± 7.6	299 ± 5.8	75.1 ± 2.62	67.2 ± 2.00
6	328 ± 6.0	319 ± 5.9	70.7 ± 2.43	69.9 ± 2.51
7	358 ± 9.1	346 ± 7.7	74.0 ± 1.11	67.6 ± 1.16
8	361 ± 8.9	358 ± 6.0	78.7 ± 2.49	69.3 ± 2.23
9	370 ± 8.8	371 ± 7.4	80.5 ± 0.81	67.7 ± 0.80
10	383 ± 9.4	380 ± 7.5	78.1 ± 1.56	63.9 ± 1.84
11	392 ± 9.6	381 ± 7.4	76.8 ± 0.76	66.4 ± 2.36
12	408 ± 9.6	391 ± 8.0	72.6 ± 1.22	65.1 ± 0.77

**Table 2:** Metabolic parameters of ethanol and control rats.

Group	Control	Ethanol
Fasting blood glucose (mmol·L^−1^)	4.31 ± 0.32	5.12 ± 0.25**
Fasting plasma insulin (mIU·L^−1^)	18.7 ± 2.56	12.1 ± 1.13*
Total cholesterol (mmol·L^−1^)	2.27 ± 0.07	2.57 ± 0.06*
Triglycerides (mmol·L^−1^)	1.54 ± 0.09	1.84 ± 0.07*
Alanine aminotransferase (U·L^−1^)	6.39 ± 0.04	9.98 ± 0.06**
Aspartate aminotransferase (U·L^−1^)	4.36 ± 0.03	7.54 ± 0.06**

Data are expressed as the mean ± S.E.M (*n* = 20).

*P < 0.05, **P < 0.01 ethanol versus control.
